# Assessment of Biochemical Composition, Mineral Content, and Fatty Acid Profile in Maize (*Zea mays*) Cultivars Under Water Stress and Excessive Water Using Biplot Analysis

**DOI:** 10.3390/foods14081432

**Published:** 2025-04-21

**Authors:** Beyza Ciftci, Ihsan Serkan Varol, Engin Kaymaz, Sevgi Saylak, Mahmut Kaplan

**Affiliations:** 1 Department of Field Crops, Faculty of Agriculture, University of Erciyes, Kayseri 38030, Türkiye; beyzacftc.58@gmail.com (B.C.); mahmutkaplan5@hotmail.com (M.K.); 2 Department of Biosystems Engineering, Faculty of Agriculture, University of Erciyes, Kayseri 38030, Türkiye; 3 Department of Research and Development, Kayseri Sugar Factories, Kayseri 38080, Türkiye; enginks7@outlook.com; 4 GAP International Agricultural Research and Training Center, Diyarbakir 63040, Türkiye; scgsaylak@gmail.com

**Keywords:** maize, irrigation, starch, mineral, fatty acids, biplot

## Abstract

Understanding the impact of irrigation levels on maize (*Zea mays* L.) nutritional properties is crucial for optimizing water use in sustainable agriculture. This study investigates the effects of three irrigation levels (I75: 75%, I100: 100%, and I125: 125% of depleted water from field capacity) on the biochemical composition, mineral content, and fatty acid profile of five maize cultivars’ grain. Biplot analysis was employed to identify superior irrigation levels and cultivars regarding nutritional traits and to visually interpret their interrelationships. The findings indicate that increased irrigation enhances oil, protein, ash, total starch, amylopectin, resistant starch, and non-resistant starch while reducing dietary fiber, phytic acid, and amylose levels. Mineral contents generally increased with irrigation, except for sulfur, which declined, and potassium, which peaked at I100 before decreasing. The fatty acid composition was largely cultivar-dependent, with no significant effect from irrigation. Among the tested irrigation levels, I100 was the most optimal, providing the best nutritional quality and mineral composition balance across the maize cultivars. Pioneer PR31Y43 and Syngenta Dracma cultivars stood out under limited irrigation conditions (I75), Pioneer PR31G98 and Tareks OSSK644 cultivars under optimum irrigation conditions (I100), and Syngenta Inove and Tareks OSSK644 cultivars under over-irrigation conditions (I125). These results highlight the necessity of cultivar-specific irrigation strategies to maximize maize nutritional quality and resource efficiency.

## 1. Introduction

Maize (*Zea mays* L.) with a high grain yield and dry matter production per unit area is a C4 plant and has an important place among the grains that are widely grown and cultivated throughout the world [1]. Therefore, it is considered the ‘queen of cereals’ [2]. It is a staple cereal globally, used both in human nutrition and animal feeding. It is known as the third most important grain in the world after wheat and rice [3]. Although it grows in hot and humid regions, it has a wide growing area due to the cultivar of the species [4].

Various factors such as irrigation, planting time, cultivar, and fertilization significantly affect yield and quality traits in maize cultivation [5]. Soil moisture, seed viability, and temperature play a crucial role in plant growth. In sustainable agriculture, irrigation water is of great importance for achieving high-quality production [6]. Optimum irrigation is defined as providing irrigation water to the crop rootzone with the highest efficiency without generating water stress [7]. Water planning is a technique used to deliver water to the crop at the right time and in a precise manner [8]. Water stress is a limiting factor for plant growth, yield, and quality [9]. Improved water planning is crucial for enhancing water use efficiency, increasing product yield, and improving product quality [10].

Maize has several important morphological and nutritional properties, such as ripening period, ear characteristics, grain structure, grain color, resistance to pests and diseases, cold and heat, yield, and nutritional composition (starch, protein, fat, and minerals) [11]. It has a wide range of potential uses [12]; however, water stress can have various negative effects on maize crops, including grain shrinkage, decreased leaf area, and deterioration of physiological processes [13]. These factors ultimately lead to a decrease in biological efficiency and grain yield [14]. Therefore, it is important to irrigate the crops to support their growth, protection, and overall increase in yield [15].

It was reported that the biochemical properties of maize are significantly affected by growing conditions [16]. Further research has also indicated significant effects of biotic and abiotic stressors on seed quality parameters, including starch, protein, lipid, vitamin, and mineral contents [17]. However, it is known that different maize cultivars may react differently to stress conditions. Regarding food safety, it is crucial to select the appropriate cultivars that meet food and industrial requirements based on specific stress conditions [18]. Biplot analysis visually evaluates data and is widely used in agriculture. A biplot is a two-way table design that graphically displays row and column factors. In this analysis method, row and column factors show both individual relationships and binary interactions. In the Biplot analysis method, the graphical display of many traits of genotypes or applications allows visual comparison of the relationships between many genotypes or applications and many traits [19]. Therefore, the study aimed to determine the effects of different irrigation levels on the nutritional properties of maize cultivars.

## 2. Materials and Methods

### 2.1. Field Experiments

Experiments were conducted in 2022 at the trial fields of Erciyes University, Kayseri, Turkiye. Five different hybrid maize cultivars (Syngenta Inove, Syngenta Dracma (Basel, Switzerland), Tareks OSSK644 (Balikesir, Türkiye), Pioneer PR31G98, and Pioneer PR31Y43 (Johnston, IA, USA) were sown at 70 × 15 cm (row spacing × on-row plant spacing) spacing in 5 × 4.2 m plots. The experimental design was split-split plots with 3 replicates. One deficit and one excessive irrigation treatment (I75, I100, and I125) were generated. In I75 (25% deficit), 75% of the depleted water from the field capacity was provided. In I100 (full irrigation), 100% of the depleted water from field capacity was provided. In I125 (25% excessive), 125% of the depleted water was provided. Drip irrigation was practiced, and a water meter was placed to determine the water to be given to each plot. Irrigation treatments were placed in main plots, and maize cultivars were placed in sub-plots. About 100 kg/ha P_2_O_5_ and 125 kg/ha N were also supplied at sowing, and 125 kg/ha N was supplied at a plant height of about 50 cm. Weed control was practiced by herbicide after emergence. Plants were harvested at the physiological maturity stage, and grains were threshed.

### 2.2. Soil and Climate Characteristics

Soil sampling was practiced from 0 to 120 cm soil profile (in 30 cm intervals) before sowing in 2022. Samples were then analyzed for different physical and chemical properties. Soils were sandy-loam in texture, slightly alkaline, poor in organic matter, and rich in K and P.

Mean temperature was 19.9 °C, mean relative humidity was 49.1%, and total rainfall in the May–September period was 129.7 mm. The experimental year had similar relative humidity, temperatures, and rainfall with long-term (1969–2021) averages.

### 2.3. Biochemical Assays

Maize grain samples were dried at 60 °C. Dried grain samples were then ground to make them ready for biochemical analyses. All biochemical analyses were performed in triplicate.

Crude protein: Sample N contents were determined with the use of the Kjeldahl method, and the N × 6.25 formula was used to calculate the crude protein content of the samples [20].

Crude ash: Sample crude ash contents were determined through ashing about 1 g of sample at 550 °C for 8 h [20].

Crude oil: Seed samples (3 g) were dissolved with ether in a Soxhlet cartridge. Oil-extracted samples were then kept in a drying chamber at 95 °C for an hour, cooled in a desiccator, and ether extract values were calculated with the aid of the following equation [20].

Crude oil % = (Crude oil weight/sample weight) × 100

Resistant, non-resistant, and total starch content: Megazyme Resistant Starch Assay kits (K-RSTAR, Megazyme International Ireland Ltd. Co., Wicklow, Ireland) were used to obtain different starch components (resistant, non-resistant, and total).

Amylose and amylopectin content: Megazyme Amylose/Amylopectin Analysis Kits (K-AMYL, Megazyme International Ireland, Wicklow, Ireland) were used to determine amylose and amylopectin fractions of the starch.

Pythic acid: Myo-inositol assay kits (K-INOSL, Megazyme Intl, Wicklow, Ireland) were used to determine phytic acid contents.

Dietary fiber: Total dietary fiber analysis of the samples was performed using the Megazyme total dietary fiber kit (K-TDFR, Megazyme Intl, Wicklow, Ireland) and was calculated as % dietary fiber content.

### 2.4. Mineral Contents

For mineral contents, maize samples (0.5 g) were acid-digested in 10 mL of nitric + perchloric acid mixture. Resultant solutions were diluted with distilled water, and mineral (Ca, Na, K, S, Mg, P, Cu, Fe, Zn, and Mn) readings were performed in a spectrophotometer (Agilent 5800 ICP-OES, Santa Clara, CA, USA) [21].

### 2.5. Fatty Acids Composition

Fatty acids from the lipid extracts were transformed into methyl esters using a 2% sulfuric acid (*v*/*v*) solution in methanol. The resulting fatty acid methyl esters (FAMEs) were then extracted with n-hexane. Separation and quantification of the methyl esters were carried out using gas chromatography with flame ionization detection (Shimadzu GC, 17 Ver.3, Kyoto, Japan), connected to a glass GC 10 software computing recorder. The chromatographic analysis was conducted on a capillary column (25 m in length, 0.25 mm in diameter, Permabound 25, Machery-Nagel, Düren, Germany) with nitrogen as the carrier gas at a flow rate of 0.8 mL/min. The temperatures for the column, detector, and injector valve were set between 130 and 220 °C and 240–280 °C, respectively. Individual compounds were identified by comparing their retention times with those of authentic standard mixtures analyzed under identical conditions [16,22].

### 2.6. Static Analysis

Data were passed through a variance analysis [23]. Significant means were then compared with an LSD test. Using Biplot analysis, irrigation variety and trait Biplot plots were created to visually present the changes in nutritional properties of maize cultivars at different irrigation levels [24].

## 3. Results

### 3.1. Biochemical Parameters

Irrigation treatments and cultivars had significant effects on kernel amylopectin levels (*p* < 0.01). For the other biochemical characteristics, irrigation, cultivar, and irrigation × cultivar interactions resulted in significant differences at the *p* < 0.01 level.

Crude protein contents increased with increasing irrigation water levels. The greatest values were seen in IE125 treatments of Tareks OSSK644 cultivar (11.91%) and the least in I75 treatment of Pioneer PR31Y43 cultivar (7.70%). For crude oil contents, the lowest value was obtained from I75 treatment of Pioneer PR31G98 cultivar (2.6%), and the highest value was obtained from I125 treatment of Pioneer PR31Y43 cultivar (3.87%). While the lowest crude oil values were obtained from I75 treatments in which 25% water deficit was applied, the highest values were obtained from I125 treatments in which 25% excess water was applied. I100 and I125 treatments were included in the same statistical group. For crude oil content of the cultivars, the Pioneer PR31G98 cultivar had the lowest crude oil value (2.99%), while there was no significant difference between the other cultivars. Dietary fiber levels decreased with increasing irrigation quantities, and the present values ranged from 5.66% (I75) to 2.72% (I125). The highest value was obtained from the I75 treatment of Syngenta Inove cultivar (7.62%) and the least in I125 treatments of Pioneer PR31Y43 cultivar (2.21%). Phytic acid values ranged from 1.61% (Pioneer PR31Y43—I75) to 0.92% (Syngenta Dracma—I125). While the Pioneer PR31Y43 cultivar had the highest phytic acid content, phytic acid levels decreased with increasing irrigation water quantities. Amylose contents also had similar trends. The highest amylose value was obtained from the I75 treatment of Tareks OSSK644 cultivar (37.29%) and the least from the I125 treatments of Syngenta Dracma cultivar (26.63%). Unlike amylose, amylopectin contents increased with increasing irrigation water quantities. The lowest values were seen in I75 treatments (65.67%). I100 and I125 treatments were placed into the same statistical group (70.84% and 70.94%, respectively). While Syngenta Inove cultivar had the highest amylopectin content (70.89%), Tareks OSSK644 cultivar had the lowest value (66.72%). Resistant starch (RS) values varied between 0.40% (Tareks OSSK644—I125) and 0.08% (Pioneer PR31Y43—I75). The lowest non-resistant starch (NRS) value was obtained from I75 treatment of Pioneer PR31Y43 cultivar (59.96%), and the highest from I125 treatment of Syngenta Dracma cultivar (78.13%). Total starch (TS) values ranged from 60.03% (Pioneer PR31Y43—I75) to 78.41% (Syngenta Dracma—I125). RS, NRS, and TS of all cultivars increased with increasing irrigation water quantities. Crude ash levels had a positive linear relationship with irrigation water levels. The lowest crude ash contents were seen in I75 treatment of Pioneer PR31Y43 cultivar (1.36%), and the highest value was obtained from I125 treatment of Syngenta Inove cultivar (2.60%) (Table 1).

### 3.2. Mineral Contents

Findings related to mineral content are given in Table 2. Irrigation treatments, cultivars, and irrigation × cultivar interactions had significant effects on mineral contents (*p* < 0.01). The highest Ca content was obtained from I100 treatment of Syngenta Inove cultivar (271.52 ppm) and the lowest from I125 of Syngenta Dracma cultivar (74.17 ppm). While the greatest values were seen in I100 treatments, the lowest average Ca values were obtained from I125 treatments in which 25% excess water was applied. Na contents varied between 320.96 (Syngenta Inove—I125) and 142.28 ppm (Syngenta Inove—I75). Na contents increased with increasing irrigation water levels. Like Ca, K values also decreased with excessive irrigation. The highest K content was obtained from I100 treatment of Syngenta Dracma cultivar (3814.09 ppm) and the lowest from I125 treatment of Syngenta Inove cultivar (1261.90 ppm). S values decreased with increasing irrigation water quantities. The highest S contents were seen in I75 treatment of Syngenta Dracma cultivar (951.53 ppm) and the lowest in I125 treatment of Syngenta Inove cultivar (448.58 ppm). Mg contents varied between 593.06 ppm (Syngenta Inove—I75) and 1395.73 ppm (Syngenta Dracma—I100). P contents increased with increasing irrigation water quantities. The highest P contents were seen in I125 treatment of Syngenta Dracma cultivar (4140.64 ppm) and the lowest in I75 treatment of Syngenta Inove cultivar (1731.43 ppm). The highest Cu content was obtained from I75 treatment of Syngenta Inove cultivar (9.02 ppm) and the lowest from I125 treatment of Tareks OSSK644 cultivar (2.95 ppm). The greatest Fe contents were seen as 19.17 ppm (Pioneer PR31G98—I100), while the least was 4.74 ppm (Pioneer PR31G98—I75). The lowest Mn content was determined as 4.08 ppm (I75—Syngenta Inove cultivar) and the highest as 11.41 ppm (I125—Syngenta Dracma cultivar). Cu, Fe, and Mn contents increased with increasing irrigation water quantities. Like other minerals, Zn was also significantly affected by both irrigation treatments and maize cultivars. Zinc contents varied between 9.02 ppm (I75—Syngenta Inove cultivar) and 32.52 ppm (I100—Pioneer PR31G98 cultivar) (Table 2).

### 3.3. Fatty Acid Composition

While oleic and linoleic acid were not affected by water stress, there were significant differences (*p* < 0.01) among the cultivars. The lowest oleic acid ratios were seen in the Syngenta Dracma cultivar (27.65%) and the greatest in the Tareks OSSK644 cultivar (29.65%). Average linoleic acid ratios varied between 58.90% (Syngenta Inove cultivar) and 54.84% (Pioneer PR31Y43 cultivar). Irrigation treatments, cultivars, and irrigation × cultivar interactions had significant effects on myristic, stearic, and linolenic acid ratios. The lowest myristic acid content was obtained from the I75 treatment of Tareks OSSK644 cultivar (0.06%) and the highest from the I100 treatment of Pioneer PR31Y43 cultivar (0.09%). The least stearic acid levels were seen in I75 treatments of Syngenta Inove cultivar (1.62%) and the highest in I100 treatment of Pioneer PR31G98 cultivar (2.50%). Linolenic acid levels increased with increasing irrigation water quantities, but I100 and I125 treatments were placed into the same statistical group. While the greatest linolenic acid levels were obtained from I100 treatment of Pioneer PR31Y43 cultivar (2.29%), the lowest linolenic acid ratio was obtained from I75 treatment of Syngenta Inove cultivar (1.40%). While palmitic acid ratios were not affected by irrigation water levels, effects of cultivars and irrigation × cultivar interactions were found to be significant (*p* < 0.01). Palmitic acid levels ranged from 10.58% (Tareks OSSK644—I125) and 12.63% (Pioneer PR31Y43—I125) (Table 3).

### 3.4. Relationship Among Biochemical Parameters, Mineral and Fatty Acid Profile in Irrigation and Maize Cultivars

**Figure 1:**
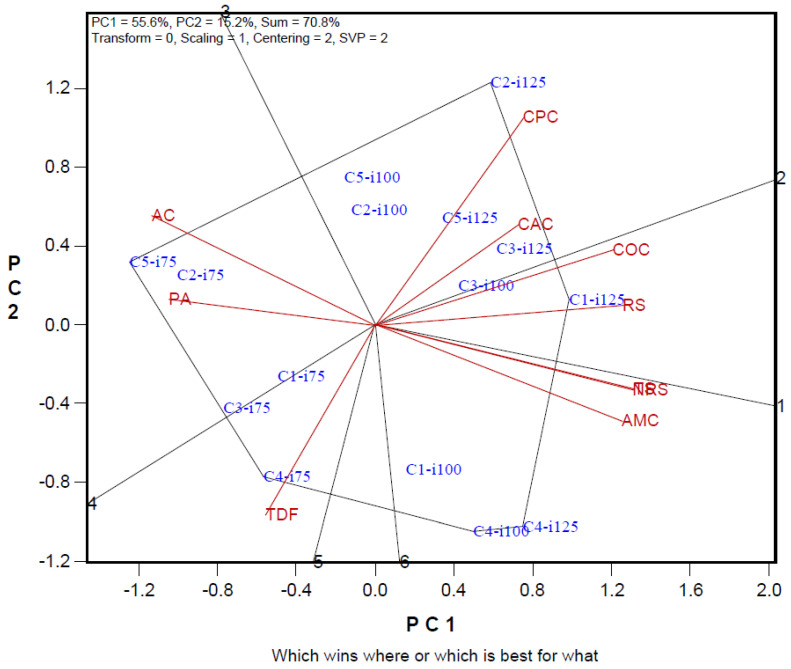
Polygon views of the biplot based on symmetrical scaling for the which-won-what pattern for irrigation maize cultivars and biochemical parameters. C1: Syngenta Inove; C2: Tareks OSSK644; C3: Pioneer PR31G98; C4: Syngenta Dracma; C5: Pioneer PR31Y43; i75: I75-75%; i100: I100-100%; i125: I125-125%; COC: crude oil; CPC: crude protein; TDF: total dietary fiber; PA: phytic acid; AC: amylose; AMC: amylopectin; RS: resistant starch; NRS: non-resistant starch; TS: total starch; CAC: crude ash.

Irrigation–cultivars–biochemical analysis biplot explained 70.8% of total variation (PC1 55.6% and PC2 15.2%). Among the examined biochemical parameters, the properties with the lowest separation power were amylose and phytic acid. While a positive relationship was found between amylopectin, non-resistant starch, and total starch in the examined properties, a negative relationship was observed between these parameters and amylose. A negative relationship was determined between crude protein content and total dietary fiber. In the I125 irrigation application, Syngenta Inove, Tareks OSSK644, and Pioneer PR31G98 cultivars came to the forefront in terms of crude ash, crude fat, and resistant starch. In the I75 irrigation level, all maize cultivars formed a group in terms of total dietary fiber, phytic acid, and amylose properties (Figure 1).

**Figure 2:**
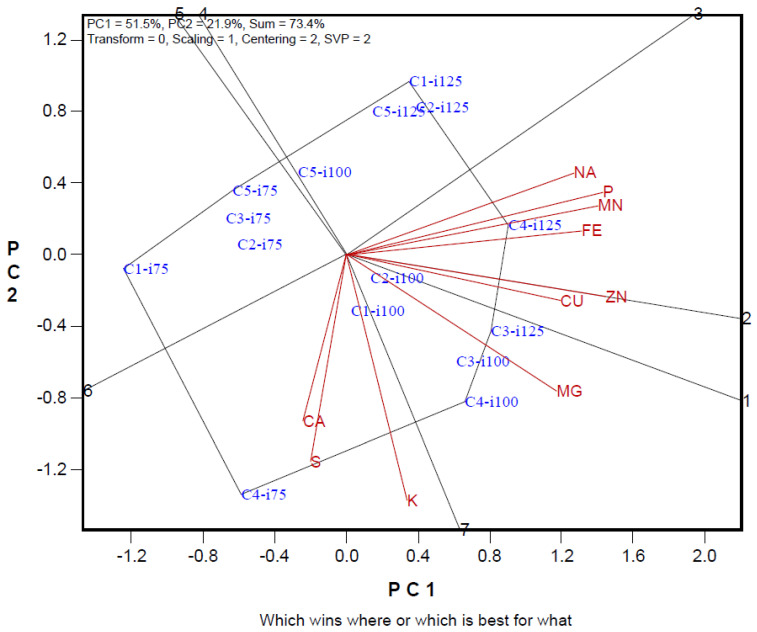
Polygon views of the biplot based on symmetrical scaling for the which-won-what pattern for irrigation maize cultivars and mineral contents. C1: Syngenta Inove; C2: Tareks OSSK644; C3: Pioneer PR31G98; C4: Syngenta Dracma C5: Pioneer PR31Y43; i75: I75-75%; i100: I100-100%; i125: I125-125%.

Biplot analysis was used to compare the mineral content of maize cultivars at different irrigation levels and to create groups in terms of irrigation-cultivars and mineral content (Figure 2). Biplot analysis explained 73.4% of the total variation in mineral content data. In the study examining the effect of irrigation on mineral content in maize cultivars, the separation power of Ca and Cu elements was low. The separation power of other elements was similar. Positive relationships were determined between Ca and S, Cu and Zn, Na, Fe, Mn, and P from mineral substances. While the mineral content in maize cultivars increased at I100 and I125 irrigation levels, the I75 irrigation level and Syngenta Dracma maize variety came to the forefront in terms of Ca and S content. Tareks OSSK644 and Pioneer PR31Y43 cultivars were observed to have low levels of mineral content.

**Figure 3:**
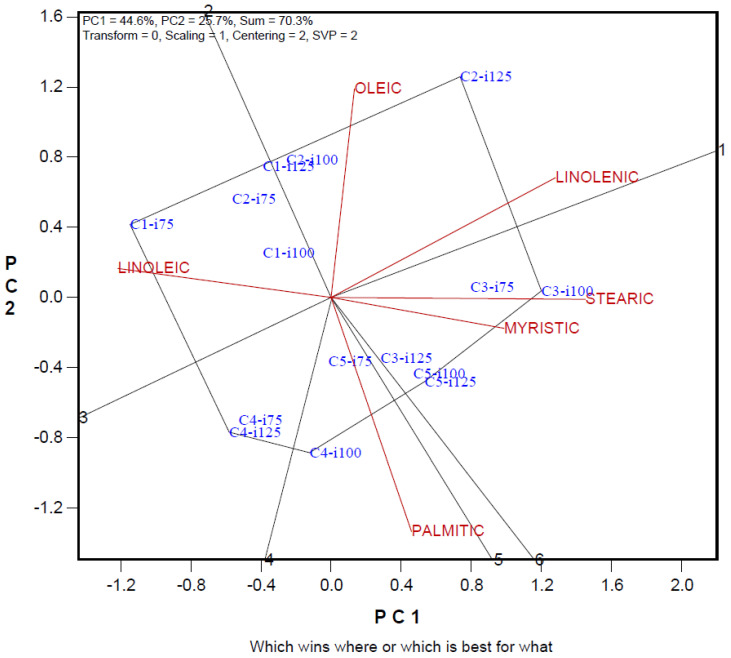
Polygon views of the biplot based on symmetrical scaling for the which-won-what pattern for irrigation maize cultivars and fatty acid composition. C1: Syngenta Inove; C2: Tareks OSSK644; C3: Pioneer PR31G98; C4: Syngenta Dracma; C5: Pioneer PR31Y43; i75: I75-75%; i100: I100-100%; i125: I125-125%.

The relationships between fatty acid composition of maize cultivars grown at different irrigation levels, the Biplot prepared to determine the prominent irrigation levels and cultivars, are given in Figure 3. While the separation power of the variation in myristic acid content was low, the separation power of oleic and palmitic acids was high. While a positive relationship was determined between myristic and stearic acids, a negative relationship was determined between these fatty acids and linoleic acid. Pioneer PR31G98 variety stood out in terms of myristic and stearic content at I75 and I100 irrigation levels, Syngenta Dracma cultivar stood out in terms of palmitic acid content at I100 irrigation levels and Pioneer PR31Y43 cultivar stood out in terms of palmitic acid content at I100 and I125 irrigation levels, and Syngenta Inove cultivar stood out in terms of linoleic acid content at I75 irrigation level.

## 4. Discussion

Maize is an important source of starch, oil, and minerals, especially in developing countries. The quality, chemical composition, and mineral concentration of maize kernels are influenced by genetic characteristics, ecological conditions, and agricultural practices [25,26]. Under water stress conditions, changes in assimilate transportation cause variations in metabolic and enzyme activities, leading to modifications in seed chemical composition [10]. Water stress leads to an increase in cell wall components, resulting in higher fiber content in the kernel while concurrently reducing the ash, protein, oil, and starch content [9,27]. The limited availability of water hinders the transportation of nutrients, resulting in a reduction in protein and starch content in grains. A large portion of nitrogen transported from the vegetative organs with sufficient water after flowering contributes to the increased nitrogen content in the grain. Post-flowering water stress significantly affects the biochemical composition of grains [16]. As reported by other researchers in the current study, water stress caused a decrease in ash, protein, starch, and fat content while increasing fiber content.

Irrigation treatments had a significant impact on kernel starch contents. The increase in irrigation leads to an increase in starch biosynthesis due to the prolonged senescence of plants and/or the grain filling period. Drought stress affects starch, amylose, and amylopectin contents [28]. According to Kaplan et al. [18], as irrigation increases, the ratio of resistant starch and amylopectin increases while the amount of amylose decreases. Similarly, Yu et al. [28] indicated that water limitation decreased the total starch and amylose content as well as the amylose/amylopectin ratio of barley grains. Conversely, Wang et al. [29] worked on wheat and stated that irrigation increased amylopectin and starch contents but decreased amylose/amylopectin ratios. In the current study, water stress decreased the amylose/amylopectin ratio.

In line with this study, several researchers have suggested that the amount of irrigation has a significant effect on the oil content of maize kernels. Ali et al. [17] discovered that low irrigation levels led to a significant reduction in oil content. Liu et al. [30] and Kaplan et al. [16] found no difference in kernel oil content of maize grown with varying levels of irrigation. Additionally, Da Ge et al. [31] stated that moderate drought increased the oil content, while severe drought reduced it. Since maize oil is a hereditary trait and genetic factors have a greater impact than the external environment, different findings were reported in these studies [32].

Oleic and linoleic acids constitute the major fatty acids of maize kernels. Akram et al. [33] reported a negative correlation between increasing linoleic acid contents and decreasing oleic acid contents. In this study, although irrigation did not significantly affect these fatty acids, the aforementioned negative correlation was observed. Pavlista et al. [34] stated that the amount of linolenic acid, as the primary component, increased with irrigation, while irrigation did not alter the fatty acid profiles of canola seeds significantly. Similarly, there was no significant effect of maize varieties on fatty acid composition.

In cases where the soil is severely lacking water, the mobility of ions can be reduced. This occurs when air replaces water in pore spaces, causing soil curvature and increased retention force by soil colloids. As a consequence, nutrient absorption decreases, ultimately leading to insufficient plant needs [35]. On the other hand, watering plants can enhance the mobility and availability of less mobile nutrients like P and K [16]. In a research carried out by Da Ge et al. [31], in plants subjected to water stress, a significant reduction of phosphorus (P) and potassium (K) levels was observed. Similarly, Kresović et al. [32] found that the highest concentration of potassium was achieved through full irrigation application, which also led to a significant increase in magnesium (Mg) concentration. Maintaining optimal soil moisture within the root zone can enhance the mobility of micronutrients. Grain Zn and Cu levels vary significantly with irrigation levels. Soil moisture is the key factor that facilitates the diffusion of adequate Zn to the roots. Thus, it plays a critical role in soils where Zn availability is low [36]. As reported by the other researchers, the mineral matter content of the maize varieties in the study increased with irrigation.

The rate of explaining the variation we obtained in the biplot chart was high and above the reliability value [37,38]. In the biplot graph, traits with short vector lengths are defined as traits with low discrimination power in explaining the variation. The trait with a long vector has a high capacity for distinguishing genotypes or applications [24,39]. In the study, it plays an important role in determining the effects of irrigation practices and variety differences on the examined traits in corn cultivars grown at different irrigation levels. There is a very close relationship between the cosine value of the angle between two trait vectors on the biplot graph and these traits. Traits with close angles on the biplot graph are positively related to each other. With biplot analysis, the most suitable irrigation level and variety screening can be carried out in terms of desired traits, and the best irrigation level or cultivars can be determined by the examined traits [40,41]. The polygon formed by combining the practices farthest from the center of the biplot and the lines drawn perpendicular to these polygon lines are evaluated differently from the other treatment group [24]. In our study, it was observed that the genotypes found in the diagonals were generally in the group with the highest values in terms of the traits in the same region [42].

## 5. Conclusions

Experiments were conducted to determine the effects of irrigation water quantities on the nutritional properties (protein, oil, fatty acids, starch, dietary fiber, phytic acid, and minerals) of various maize cultivars. Present findings revealed that irrigation increased oil, protein, ash, total starch, amylopectin, resistant and non-resistant starch levels, but reduced dietary fiber, phytic acid, and amylose contents. Mineral contents, except for S and K, increased with increasing irrigation levels. S contents decreased with irrigation, and K contents initially increased, then decreased. The highest K content was obtained from I100 (full irrigation) treatments. Oleic, linoleic, and palmitic fatty acid contents were not affected by irrigation, although the cultivars differed in terms of fatty acid profiles. Increasing irrigation levels increased the nutritional properties of maize kernels; however, cultivar response to irrigation varied significantly. Although irrigation levels did not significantly alter the major fatty acid composition, cultivar-specific differences were observed, emphasizing the importance of selecting maize cultivars with desirable fatty acid profiles for specific nutritional and industrial applications. Optimum irrigation (I100) is the most balanced irrigation level, maximizing protein, oil, total starch, and mineral content in all cultivars. Pioneer PR31Y43 and Syngenta Dracma should be preferred as they maintained higher fiber and amylose levels under limited water conditions. Pioneer PR31G98 and Tareks OSSK644 stood out for their high protein and starch accumulation, making them ideal choices for achieving superior nutritional quality in well-managed irrigation systems. At high irrigation levels, mineral accumulation increased except for potassium and sulfur, which decreased. Syngenta Inove and Tareks OSSK644 performed well under excessive irrigation, particularly in crude ash, protein, and resistant starch. However, excessive irrigation may result in inefficient water use without additional benefits to fatty acid composition.

## Figures and Tables

**Table 1:** Biochemical properties of maize cultivars under different irrigation levels.

	Crude Oil					Crude Protein			
Cultivars	I75	I100	I125	Means	Cultivars	I75	I100	I125	Means
Syngenta Inove	3.11 ^cd^	3.23 ^bc^	3.50 ^ab^	3.28 ^a^	Syngenta Inove	9.15 ^cd^	9.26 ^c^	9.35 ^c^	9.25 ^b^
Tareks OSSK644	2.58 ^e^	3.31 ^abc^	3.56 ^a^	3.15 ^a^	Tareks OSSK644	7.78 ^e^	10.36 ^b^	11.91 ^a^	10.02 ^a^
Pioneer PR31G98	2.16 ^f^	3.56 ^a^	3.24 ^bc^	2.99 ^b^	Pioneer PR31G98	8.09 ^e^	9.47 ^c^	10.40 ^b^	9.32 ^b^
Syngenta Dracma	2.81 ^de^	3.42 ^ab^	3.23 ^bc^	3.15 ^a^	Syngenta Dracma	7.71 ^e^	7.63 ^e^	8.67 ^d^	8.01 ^c^
Pioneer PR31Y43	2.59 ^e^	3.29 ^abc^	3.57 ^a^	3.15 ^a^	Pioneer PR31Y43	7.70 ^e^	10.31 ^b^	11.54 ^a^	9.85 ^a^
Means	2.65 ^b^	3.36 ^a^	3.42 ^a^		Means	8.08 ^c^	9.41 ^b^	10.37 ^a^	
	Total Dietary Fiber					Phytic Acid			
Cultivars	I75	I100	I125	Means	Cultivars	I75	I100	I125	Means
Syngenta Inove	7.62 ^a^	7.37 ^a^	2.40 ^ij^	5.80 ^a^	Syngenta Inove	1.29 ^b^	1.17 ^c^	1.06 ^d^	1.17 ^b^
Tareks OSSK644	3.61 ^de^	2.81 ^gh^	2.22 ^ij^	2.88 ^d^	Tareks OSSK644	1.57 ^a^	0.96 ^de^	0.94 ^e^	1.15 ^b^
Pioneer PR31G98	6.41 ^b^	4.28 ^c^	3.01 ^fg^	4.56 ^c^	Pioneer PR31G98	1.31 ^b^	1.22 ^bc^	0.54 ^f^	1.03 ^c^
Syngenta Dracma	7.32 ^a^	4.22 ^c^	3.74 ^d^	5.09 ^b^	Syngenta Dracma	0.99 ^de^	0.98 ^de^	0.92 ^e^	0.96 ^d^
Pioneer PR31Y43	3.34 ^ef^	2.60 ^hi^	2.21 ^j^	2.71 ^d^	Pioneer PR31Y43	1.61 ^a^	1.26 ^bc^	1.25 ^bc^	1.37 ^a^
Means	5.66 ^a^	4.26 ^b^	2.72 ^c^		Means	1.35 ^a^	1.12 ^b^	0.94 ^c^	
	Amylose					Amylopectin			
Cultivars	I75	I100	I125	Means	Cultivars	I75	I100	I125	Means
Syngenta Inove	32.78 ^cde^	28.61 ^fgh^	27.21 ^gh^	29.53 ^b^	Syngenta Inove	67.51	72.41	72.74	70.89 ^a^
Tareks OSSK644	37.29 ^a^	32.43 ^cde^	31.75 ^de^	33.82 ^a^	Tareks OSSK644	62.97	67.83	69.35	66.72 ^b^
Pioneer PR31G98	33.68 ^bcd^	34.81 ^abc^	32.21 ^cde^	33.56 ^a^	Pioneer PR31G98	66.43	72.13	68.08	68.88 ^ab^
Syngenta Dracma	33.55 ^bcd^	27.65 ^gh^	26.63 ^h^	29.28 ^b^	Syngenta Dracma	66.69	72.90	74.39	71.33 ^a^
Pioneer PR31Y43	36.36 ^ab^	31.18 ^def^	30.11 ^efg^	32.55 ^a^	Pioneer PR31Y43	64.74	68.93	70.16	67.94 ^b^
Means	34.73 ^a^	30.94 ^b^	29.58 ^c^		Means	65.67 ^b^	70.84 ^a^	70.94 ^a^	
	Resistant Starch					Non-Resistant Starch			
Cultivars	I75	I100	I125	Means	Cultivars	I75	I100	I125	Means
Syngenta Inove	0.16 ^e^	0.23 ^d^	0.37 ^b^	0.25 ^a^	Syngenta Inove	65.20 ^de^	69.75 ^bcd^	76.21 ^ab^	70.39 ^b^
Tareks OSSK644	0.12 ^f^	0.15 ^e^	0.40 ^a^	0.22 ^b^	Tareks OSSK644	66.11 ^de^	66.89 ^d^	69.13 ^cd^	67.37 ^c^
Pioneer PR31G98	0.15 ^e^	0.26 ^c^	0.26 ^c^	0.23 ^b^	Pioneer PR31G98	65.71 ^de^	74.98 ^abc^	76.20 ^ab^	72.30 ^ab^
Syngenta Dracma	0.16 ^e^	0.26 ^c^	0.28 ^c^	0.23 ^b^	Syngenta Dracma	66.62 ^d^	75.26 ^abc^	78.13 ^a^	73.33 ^a^
Pioneer PR31Y43	0.08 ^g^	0.15 ^e^	0.17 ^e^	0.13 ^c^	Pioneer PR31Y43	59.96 ^e^	65.21 ^de^	73.64 ^abc^	66.27 ^c^
Means	0.13 ^c^	0.21 ^b^	0.30 ^a^	0.21	Means	64.72 ^c^	70.42 ^b^	74.66 ^a^	
	Total Starch					Crude Ash			
Cultivars	I75	I100	I125	Means	Cultivars	I75	I100	I125	Means
Syngenta Inove	65.36 ^de^	69.98 ^bcd^	76.58 ^a^	70.64 ^a^	Syngenta Inove	1.70 ^d^	1.82 ^cd^	2.60 ^a^	2.04 ^a^
Tareks OSSK644	66.23 ^de^	67.04 ^d^	69.52 ^cd^	67.60 ^b^	Tareks OSSK644	1.50 ^e^	1.50 ^e^	2.11 ^b^	1.70 ^b^
Pioneer PR31G98	65.86 ^de^	75.24 ^abc^	76.47 ^ab^	72.52 ^a^	Pioneer PR31G98	1.89 ^c^	2.23 ^e^	2.11 ^b^	2.08 ^a^
Syngenta Dracma	66.77 ^d^	75.52 ^abc^	78.41 ^a^	73.57 ^a^	Syngenta Dracma	1.40 ^e^	1.41 ^e^	1.41 ^e^	1.41 ^d^
Pioneer PR31Y43	60.03 ^e^	65.37 ^de^	73.81 ^abc^	66.40 ^b^	Pioneer PR31Y43	1.36 ^e^	1.89 ^c^	1.42 ^e^	1.56 ^c^
Means	64.85 ^c^	70.63 ^b^	74.96 ^a^		Means	1.57 ^c^	1.77 ^b^	1.93 ^a^	

Means within columns compare different maize cultivars under the same irrigation level, while means within rows compare different irrigation levels for the same cultivar. For interactions (cultivar × irrigation level), comparisons were conducted both within rows and columns. a–j: Different lowercase letters represent significant differences.

**Table 2:** Mineral contents of maize cultivars under different irrigation levels.

Ca (ppm)					Na (ppm)				
Cultivars	I75	I100	I125	Mean	Cultivars	I75	I100	I125	Mean
Syngenta Inove	208.67 ^c^	271.52 ^a^	171.87 ^e^	217.35 ^a^	Syngenta Inove	142.28 ^e^	298.75 ^ab^	320.96 ^a^	254.00 ^b^
Tareks OSSK644	177.85 ^e^	138.67 ^f^	122.95 ^gh^	146.49 ^d^	Tareks OSSK644	279.36 ^b^	279.69 ^b^	288.41 ^b^	282.49 ^a^
Pioneer PR31G98	113.82 ^h^	188.02 ^d^	214.28 ^c^	172.04 ^b^	Pioneer PR31G98	199.93 ^cd^	289.30 ^b^	301.85 ^ab^	263.69 ^b^
Syngenta Dracma	247.77 ^b^	179.42 ^de^	74.17 ^i^	167.12 ^c^	Syngenta Dracma	143.71 ^e^	281.41 ^b^	295.61 ^ab^	240.24 ^c^
Pioneer PR31Y43	123.97 ^g^	125.45 ^g^	144.55 ^f^	131.32 ^e^	Pioneer PR31Y43	178.13 ^d^	219.28 ^c^	298.38 ^ab^	231.93 ^c^
Mean	174.42 ^b^	180.62 ^a^	145.57 ^c^		Mean	188.68 ^c^	273.69 ^b^	301.04 ^a^	
K (ppm)					S (ppm)				
Cultivars	I75	I100	I125	Mean	Cultivars	I75	I100	I125	Mean
Syngenta Inove	3063.14 ^c^	2730.96 ^de^	1261.90 ^j^	2352.00 ^c^	Syngenta Inove	694.88 ^f^	790.28 ^e^	448.58 ^i^	644.58 ^d^
Tareks OSSK644	2497.82 ^ef^	3021.65 ^c^	1623.67 ^hi^	2381.05 ^c^	Tareks OSSK644	940.78 ^ab^	808.24 ^de^	613.90 ^gh^	787.64 ^c^
Pioneer PR31G98	1831.47 ^h^	3801.85 ^a^	3700.12 ^a^	3111.15 ^b^	Pioneer PR31G98	921.08 ^ab^	876.41 ^bcd^	702.80 ^f^	833.43 ^b^
Syngenta Dracma	3404.94 ^b^	3814.09 ^a^	2898.15 ^cd^	3372.39 ^a^	Syngenta Dracma	951.53 ^a^	889.03 ^abc^	827.81 ^cde^	889.46 ^a^
Pioneer PR31Y43	2168.26 ^g^	2265.50 ^fg^	1556.02 ^i^	1996.59 ^d^	Pioneer PR31Y43	671.72 ^fg^	640.18 ^fgh^	597.94 ^h^	636.62 ^d^
Mean	2593.13 ^b^	3126.81 ^a^	2207.97 ^c^		Mean	836.00 ^a^	800.83 ^b^	638.21 ^c^	
Mg (ppm)					P (ppm)				
Cultivars	I75	I100	I125	Mean	Cultivars	I75	I100	I125	Mean
Syngenta Inove	593.06 ^j^	1116.07 ^de^	1118.05 ^de^	942.39 ^c^	Syngenta Inove	1731.43 ^g^	3431.68 ^cd^	3478.34 ^cd^	2880.48 ^b^
Tareks OSSK644	683.26 ^j^	1070.75 ^ef^	907.41 ^gh^	887.14 ^d^	Tareks OSSK644	2314.60 ^f^	3072.91 ^e^	3639.55 ^bc^	3009.02 ^b^
Pioneer PR31G98	795.76 ^i^	1108.93 ^e^	1330.66 ^ab^	1078.45 ^b^	Pioneer PR31G98	2410.95 ^f^	3966.64 ^ab^	3930.66 ^ab^	3436.08 ^a^
Syngenta Dracma	1288.61 ^bc^	1395.73 ^a^	1217.76 ^cd^	1300.70 ^a^	Syngenta Dracma	1253.28 ^h^	3401.40 ^cde^	4140.64 ^a^	2931.77 ^b^
Pioneer PR31Y43	822.31 ^hi^	856.95 ^hi^	969.80 ^fg^	883.02 ^d^	Pioneer PR31Y43	2216.07 ^f^	2515.94 ^f^	3249.98 ^de^	2660.67 ^c^
Mean	836.60 ^b^	1109.69 ^a^	1108.73 ^a^		Mean	1985.27 ^c^	3277.71 ^b^	3687.84 ^a^	
Cu (ppm)					Fe (ppm)				
Cultivars	I75	I100	I125	Mean	Cultivars	I75	I100	I125	Mean
Syngenta Inove	0.76 ^j^	1.66 ^h^	2.23 ^def^	1.55 ^d^	Syngenta Inove	9.74 ^e^	13.39 ^d^	15.42 ^c^	12.85 ^b^
Tareks OSSK644	1.14 ^i^	2.43 ^cd^	2.95 ^a^	2.17 ^b^	Tareks OSSK644	9.30 ^e^	10.57 ^e^	15.10 ^c^	11.66 ^c^
Pioneer PR31G98	1.69 ^h^	2.39 ^de^	2.65 ^bc^	2.25 ^b^	Pioneer PR31G98	4.74 ^f^	19.17 ^a^	18.22 ^ab^	14.05 ^a^
Syngenta Dracma	2.80 ^ab^	2.89 ^ab^	2.94 ^a^	2.88 ^a^	Syngenta Dracma	5.23 ^f^	17.37 ^b^	17.31 ^b^	13.30 ^b^
Pioneer PR31Y43	1.94 ^g^	2.01 ^fg^	2.16 ^efg^	2.04 ^c^	Pioneer PR31Y43	5.37 ^f^	14.83 ^cd^	14.32 ^cd^	11.51 ^c^
Mean	1.67 ^c^	2.28 ^b^	2.59 ^a^		Mean	6.88 ^c^	15.07 ^b^	16.07 ^a^	
Zn (ppm)					Mn (ppm)				
Cultivars	I75	I100	I125	Mean	Cultivars	I75	I100	I125	Mean
Syngenta Inove	9.02 ^h^	18.82 ^ef^	23.11 ^cd^	16.99 ^c^	Syngenta Inove	4.08 ^g^	7.87 ^cd^	8.64 ^bc^	6.86 ^d^
Tareks OSSK644	12.01 ^g^	24.37 ^c^	23.58 ^c^	19.99 ^b^	Tareks OSSK644	7.66 ^de^	7.84 ^cd^	9.24 ^b^	8.25 ^b^
Pioneer PR31G98	17.29 ^f^	32.52 ^a^	27.31 ^b^	25.70 ^a^	Pioneer PR31G98	6.65 ^f^	7.95 ^cd^	10.48 ^a^	8.36 ^ab^
Syngenta Dracma	17.69 ^f^	29.49 ^b^	28.94 ^b^	25.37 ^a^	Syngenta Dracma	6.05 ^f^	8.61 ^bc^	11.41 ^a^	8.69 ^a^
Pioneer PR31Y43	17.51 ^f^	18.62 ^ef^	20.72 ^de^	18.95 ^b^	Pioneer PR31Y43	6.70 ^f^	6.72 ^ef^	8.59 ^bcd^	7.33 ^c^
Mean	14.70 ^b^	24.76 ^a^	24.73 ^a^		Mean	6.23 ^c^	7.80 ^b^	9.67 ^a^	

Means within columns compare different maize cultivars under the same irrigation level, while means within rows compare different irrigation levels for the same cultivar. For interactions (cultivar × irrigation level), comparisons were conducted both within rows and columns. a–j: Different lowercase letters represent significant differences.

**Table 3:** Fatty acid composition of maize cultivars under different irrigation levels.

Myristic acid (C14:0)					Palmitic acid (C16:0)				
Cultivars	I75	I100	I125	Mean	Cultivars	I75	I100	I125	Mean
Syngenta Inove	0.08 ^bcde^	0.08 ^cde^	0.06 ^hi^	0.07 ^b^	Syngenta Inove	11.35 ^bcde^	10.78 ^de^	10.54 ^e^	10.89 ^c^
Tareks OSSK644	0.06 ^i^	0.07 ^ef^	0.09 ^abc^	0.07 ^b^	Tareks OSSK644	10.70 ^e^	10.83 ^cde^	10.58 ^e^	10.70 ^c^
Pioneer PR31G98	0.08 ^de^	0.09 ^a^	0.08 ^de^	0.08 ^a^	Pioneer PR31G98	11.74 ^abcd^	11.83 ^abc^	12.62 ^a^	12.06 ^b^
Syngenta Dracma	0.07 ^gh^	0.09 ^abcd^	0.06 ^ghi^	0.07 ^b^	Syngenta Dracma	11.79 ^abcd^	12.42 ^a^	12.08 ^ab^	12.10 ^ab^
Pioneer PR31Y43	0.07 ^fg^	0.09 ^ab^	0.09 ^abc^	0.08 ^a^	Pioneer PR31Y43	12.50 ^a^	12.54 ^a^	12.63 ^a^	12.55 ^a^
Mean	0.07 ^c^	0.08 ^a^	0.08 ^b^		Mean	11.61	11.68	11.69	
Stearic acid (C18:0)					Oleic acid (C18:1)				
Cultivars	I75	I100	I125	Mean	Cultivars	I75	I100	I125	Mean
Syngenta Inove	1.62 ^e^	1.87 ^cd^	1.94 ^c^	1.81 ^d^	Syngenta Inove	29.82	28.51	29.26	29.20 ^a^
Tareks OSSK644	1.75 ^de^	1.92 ^c^	2.17 ^b^	1.94 ^c^	Tareks OSSK644	29.05	29.76	30.15	29.65 ^a^
Pioneer PR31G98	2.30 ^b^	2.50 ^a^	1.93 ^c^	2.24 ^a^	Pioneer PR31G98	28.49	28.49	29.10	28.69 ^ab^
Syngenta Dracma	1.87 ^cd^	1.96 ^c^	1.88 ^cd^	1.90 ^c^	Syngenta Dracma	27.59	28.01	27.36	27.65 ^b^
Pioneer PR31Y43	1.99 ^c^	1.99 ^c^	2.19 ^b^	2.06 ^b^	Pioneer PR31Y43	29.17	28.91	29.12	29.07 ^a^
Mean	1.91 ^b^	2.05 ^a^	2.02 ^a^		Mean	28.83	28.74	29.00	
Linoleic acid (C18:2)					Linolenic acid (C18:3)				
Cultivars	I75	I100	I125	Mean	Cultivars	I75	I100	I125	Mean
Syngenta Inove	61.99	58.17	56.55	58.90 ^a^	Syngenta Inove	1.40 ^g^	1.70 ^bcd^	1.75 ^bc^	1.62 ^d^
Tareks OSSK644	56.15	56.76	55.47	56.13 ^bc^	Tareks OSSK644	1.71 ^bcd^	1.76 ^bc^	2.25 ^a^	1.91 ^b^
Pioneer PR31G98	55.03	55.14	54.51	54.90 ^c^	Pioneer PR31G98	2.19 ^a^	2.29 ^a^	1.81 ^b^	2.09 ^a^
Syngenta Dracma	57.19	57.51	57.79	57.50 ^ab^	Syngenta Dracma	1.47 ^fg^	1.49 ^efg^	1.62 ^de^	1.53 ^e^
Pioneer PR31Y43	55.07	54.47	54.98	54.84 ^c^	Pioneer PR31Y43	1.58 ^def^	1.81 ^b^	1.66 ^cd^	1.68 ^c^
Mean	57.09	56.41	55.86		Mean	1.67 ^b^	1.81 ^a^	1.82 ^a^	

Means within columns compare different maize cultivars under the same irrigation level, while means within rows compare different irrigation levels for the same cultivar. For interactions (cultivar × irrigation level), comparisons were conducted both within rows and columns. a–i: Different lowercase letters represent significant differences.

## Data Availability

The data presented in this study are available on request from the corresponding author. The data are not publicly available due to privacy restrictions.
